# Development and Validation of a New Anisotropic Visco-Hyperelastic Human Head Finite Element Model Capable of Predicting Multiple Brain Injuries

**DOI:** 10.3389/fbioe.2022.831595

**Published:** 2022-03-24

**Authors:** Ding Lyu, Runzhou Zhou, Chin-hsu Lin, Priya Prasad, Liying Zhang

**Affiliations:** ^1^ Department of Biomedical Engineering, Wayne State University, Detroit, MI, United States; ^2^ General Motors R&D Center, Warren, MI, United States; ^3^ Prasad Engineering, LLC, Plymouth, MI, United States

**Keywords:** finite element analysis, GHBMC human head model, brain strain validation, skull injury, facial injury, brain injury, crash induced injury index, risk function

## Abstract

This paper reports on the latest refinement of the Finite Element Global Human Body Models Consortium 50th percentile (GHBMC M50) adult male head model by the development and incorporation of a new material model into the white matter tissue of the brain. The white matter is represented by an anisotropic visco-hyperelastic material model capable of simulating direction-dependent response of the brain tissue to further improve the bio-fidelity and injury predictive capability of the model. The parameters representing the material were optimized by comparing model responses to seven experimentally reported strain responses of brains of postmortem human subjects (PMHS) subjected to head impact. The head model was subjected to rigorous validation against experimental data on force–deflection responses in the skull and face, intracranial pressure, and brain strain responses from over 34 PMHS head impact experiments. Crash-induced injury indices (CIIs) for facial bone fracture, skull fracture, cerebral contusion, acute subdural hematomas (ASDHs), and diffuse brain injury were developed by reconstructing 32 PMHS and real-world injury cases with the model. Model predicted maximum principal strain (MPS) and stress were determined as fracture CIIs for compact bone and spongy bones, respectively, in the skull and face. Brain responses in terms of MPS, MPS rates, and pressure distribution in injury producing experimental impacts were determined using the model and analyzed with logistic regression and survival analysis to develop CIIs for brain contusions, diffuse brain injuries, and ASDH. The statistical models using logistic regression and survival analysis showed high accuracy with area under the receiver operating curve greater than 0.8. Because of lack of sufficient moderate diffuse brain injury data, a statistical model was not created, but all indications are that the MPS rate is an essential brain response that discriminates between moderate and severe brain injuries. The authors stated that the current GHBMC M50 v.6.0 is an advanced tool for injury prediction and mitigation of injuries in automotive crashes, sports, recreational, and military environments.

## 1 Introduction

In the United States, approximately 2.5 million traumatic brain injury (TBI)–related emergency department visits, 280,000 TBI-related hospitalizations, and 56,000 TBI-related deaths occurred during 2013 according to the National Center for Injury Prevention and Control (Taylor et al., 2017). TBIs are commonly caused by falls (38%), exposure to mechanical forces (21%), motor vehicle crashes (20%), assaults (17%), etc., and remain a major public health problem (Feigin et al., 2013). To understand brain injury mechanisms, response, and tolerance to impact, a variety of experimental tools (e.g., sleds, pendulums, drop towers, and fluid percussion devices) have been developed. *In vivo* tests using animals and postmortem human subjects (PMHS) have been conducted to obtain the biomechanical responses relevant to injury production. Recognizing the difficulty of measuring *in vivo* strains during impact in animal or PMHS subjects, researchers have used finite element (FE) analysis to estimate deformations, strains, and strain rates in the various parts of the brain subjected to impact. These FE models continue to evolve in complexity and improve our understanding of mechanisms of brain injuries far beyond what could be learnt from experimentation.

Many human FE head models have been developed since the first simplified three-dimensional (3D) FE head model proposed by Ward and Thompson (1975). Hosey and Liu (1981) created a 3D homomorphic FE head and neck model showing basic skull and brain anatomic features with 786 elements. Ruan et al. (1994) built a well-known Wayne State University Brain Injury Model (WSUBIM) with skull and brain anatomic details by using 9,146 elements. Kang et al. (1997) developed a head model for a 50th percentile adult with 13,208 elements and validated the model with the experimental intracranial pressure as reported by Nahum et al. (1977). In their work, the material property of the brain was assumed to be linear viscoelastic and other components of the head were assumed to be elastic. Zhang et al. (2001) reported a more refined 50th percentile male human head model weighing 4.5 kg and represented by 314,500 elements. The brain material property was assumed to be linear viscoelastic. The model was validated with intracranial pressures reported by Nahum et al. (1977) and motion of the brain relative to the skull reported by Hardy et al. (2001). Twenty-four NFL player-to-player impact cases were simulated with this model, and injury thresholds for mild TBI were proposed (Zhang et al., 2004). The FE head model developed by Kleiven (2007) used an isotropic hyper-viscoelastic constitutive law to model the brain. The simulation results of mild TBI from 58 NFL cases implied that brain tissue stiffness differences in tension and compression need to be properly reflected in the model (Franceschini et al., 2006). A Simulated Injury Monitor (SIMon) head model was developed by NHTSA to simulate the anthropomorphic test dummy and American football player-to-player impacts (Takhounts et al., 2003, 2008). The injury criteria associated with the SIMon head model including maximum principal strain (MPS) and the cumulative strain damage measure (CSDM) were reported by Takhounts et al. (2013). Mao et al. (2013) developed a Global Human Body Modeling Consortium (GHBMC) M50 (50th percentile) head model and validated it against the experimental results from 35 loading cases, making it the most validated head model at that time.

Most of the head models were validated against relative skull-brain displacement and intracranial pressure data. However, strain responses of head FE models needed validation to ensure the injury-prediction based on strains and strain-based injury criterion. Hardy et al. (2007) investigated the brain strain under impact loadings at levels, which might cause brain injuries. Zhou et al. (2018) revisited the brain strain results and revised the brain strain calculation method by applying the motion histories of neutral density targets (NDTs) to the NDT triad model. Their results offer a way to increase the brain strain prediction ability of the FE brain model. Another improvement of the GHBMC FE head model would focus on incorporating the orientation of axons into the brain model to enable the calculation of axonal strains and the risk of sustaining diffuse brain injury (Giordano et al., 2014; Giordano and Kleiven, 2014; Sahoo et al., 2016). Abolfathi et al. (2009) proposed to model the anisotropic feature of brain white matter by using the multiscale micro-mechanic submodeling technologies. However, instead of using micro-mechanic submodeling methods, Zhao and Ji (2019) recommended to model the anisotropy of the white matter *via* a more cost-effective tractography-based method that was also used in our current model. The current paper reports on the latest upgrades of the GHBMC M50 head model from the prior versions. First, a new anisotropic visco-hyperelastic material model was developed and incorporated in the white matter tissue of the head model. Second, the bio-fidelity of the brain strain simulated by the model was optimized to match to MPS responses observed in PMHS tests (Zhou et al., 2018). Third, the current head model was extensively validated against responses of the skull, face, and brain at various locations in PMHS head impact tests. Last, several crash-induced injury indices (CIIs) were developed and incorporated into the model to enhance its capabilities of predicting skull fractures, facial fractures, cerebral contusion, acute subdural hematoma (ASDH), and diffuse brain injury.

## 2 Materials and Methods

### 2.1 GHBMC M50 Head Model

**FIGURE 1:**
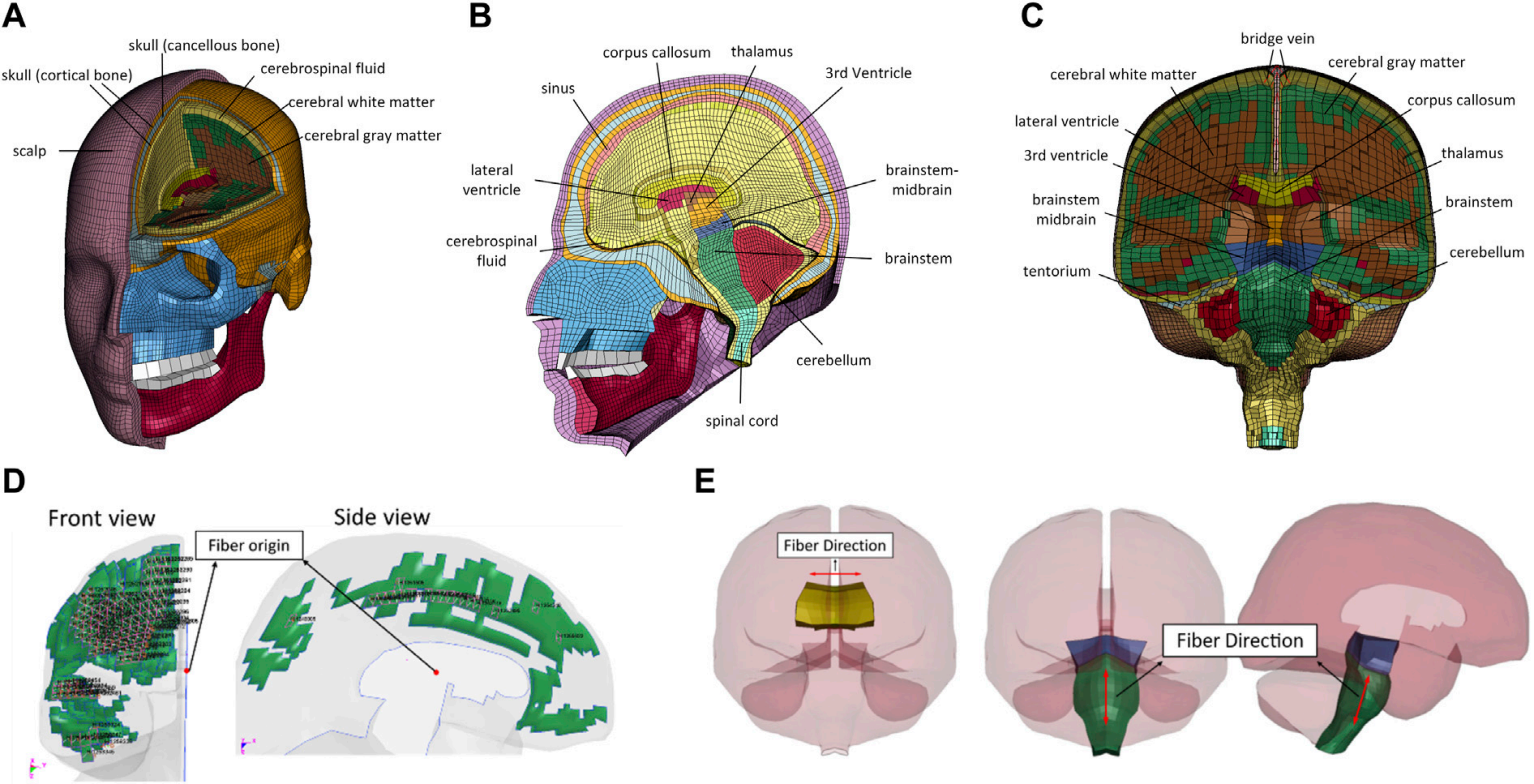
Illustration of GHBMC M50 FE head model: **(A)** frontal view; **(B)** sagittal view; **(C)** coronal view anisotropic visco-hyperelastic material model; **(D)** frontal and side views of the fiber direction defined in the subcortical white matter; **(E)** fiber directions defined in corpus callosum and brainstem.

The GHBMC M50 FE human head model is shown in Figure 1. It represents the anthropometry of a 50th percentile adult male head. The model was first developed and reported by Mao et al. (2013). The FE mesh was based on a computer-aided design dataset of the human body geometry acquired from supine MRI and CT scan techniques (Gayzik et al., 2011). The meshes of the head model were segregated explicitly to model essential anatomical components of the face, skull, and intracranial contents. The model consists of a sandwiched skull that includes outer and inner tables, diploe, scalp, various facial bones (lacrimal, maxilla, mandible, nasal, orbital, sphenoid, vomer, zygomatic, etc.), facial flesh, dura mater, arachnoid mater, pia mater, superior sagittal sinus, 11 pairs of bridging veins (BV), cerebral spinal fluid (CSF), cerebral cortex, subcortical white matter, corpus callosum, thalamus, basal ganglia, brainstem, cerebellum, lateral and third ventricles, falx, and tentorium. The current GHBMC head model v.6 consists of 246,829 elements—164,226 hexahedral solid elements, 82,581 shell elements, and 22 one-dimensional beam elements. The total mass of the FE head model is 4.6 kg. The HYPERMESH 13.0 (Altair Engineering Inc., Troy, MI, USA) and single precision LS-DYNA MPP R12.0 solver (Livermore Technology Software Corporation (LSTC), Livermore, CA, USA) were used for mesh quality improvement and simulation, respectively.

Jacobian value, warpage, aspect ratio, minimum angle, and skew of the mesh were assessed to assure appropriate mesh quality according to the criteria set for the GHBMC models. In the current model, the warpage and skew angles are less than 50° and 65° for the hexahedral and pentahedral elements, respectively. The minimum length of the element is 0.37 mm, and the aspect ratio of all elements is less than 8.0. Jacobian values of all elements were greater than 0.4, and the range of interior angles is between 25° and 160°. No mass increase at the initialization of the simulation for 0.3 μs time step is required in the current model.

Brain tissue is an ultrasoft biological material with various biomechanical characteristics under loading (Budday et al., 2019). Brain tissue is viscoelastic, and its stiffness increases with increasing strain (Chatelin et al., 2010; Budday et al., 2017). Some studies showed that brain tissue is stiffer in compression than in tension (Miller and Chinzei, 1997, 2002; Franceschini et al., 2006; Jin et al., 2013; Pogoda et al., 2014). In addition, the biomechanical response of some white matter structures is anisotropic, such as the left-right oriented corpus callosum axonal fibers and superior-inferior oriented brainstem axonal fibers (Prange and Margulies, 2002; Ning et al., 2006; Jin et al., 2013; Budday et al., 2017; Feng et al., 2017). Other studies have reported region-dependent stiffness, such as the gray matter having stiffer properties than the white matter (Budday et al., 2019; Chatelin et al., 2010; MacManus et al., 2017; Van Dommelen et al., 2010). It was assumed that the analysis results were more reliable while using more realistic material model. The above-mentioned biomechanical characteristics of the brain tissue, especially the anisotropic characteristics exhibited by the white matter, are essential properties that needed to be incorporated into the FE model to accurately predict the mechanical responses in the tissue and injuries to the brain. However, to our knowledge, none of the human or animal head FE models reported to date simulates anisotropic properties and differential compressive behavior from tensile behavior. For example, the most common viscoelastic material model is incapable of simulating differential compression or tension properties (Feng et al., 2017; Zhao and Ji, 2019). A powerful constitutive material model capable of simulating the properties under large deformation is required. The concept of hyperelasticity (a special case of Cauchy elasticity) is employed to describe the mechanical behavior of soft tissue. The basic deformation of the local kinematics may be presented by deformation gradient **F** that is denoted as

$$F=\frac{\partial x}{\partial X}\;\mathrm{or}\;F_{ij}=\frac{\partial x_{i}}{\partial X_{j}}$$ (1)

where **X** and **x** denote the Cartesian coordinates of a specific particle in the reference configuration and deformed configuration, respectively. The left Cauchy–Green deformation tensor **B** and right Green’s deformation tensor ***C*** are known as

$$\{\begin{array}{c} B=FF^{T}\;\;\;\;\;\;\;\;\;\;\;or\;\;\;\;\;\;\;\;\;B_{ij}=F_{ik}F_{kj}\\ C=F^{T}F\;\;\;\;\;\;\;\;\;\;\;or\;\;\;\;\;\;\;\;\;C_{ij}=F_{ki}F_{jk} \end{array}$$ (2)

Three principal invariants **B** are denoted as

$$I_{1}=trB,\;I_{2}=\frac{1}{2}\left[{\left(trB\right)}^{2}-trB^{2}\right],\;I_{3}=\det B$$ (3)

where “*tr*” and “det” represent the trace and determinant of the matrix, respectively. The strain energy function of hyperelastic material ***W*** may be expressed in a set of strain invariants of the left Cauchy–Green deformation tensor **B**, i.e., *W*(*I*_1_(**B**), *I*_2_(**B**), *I*_3_(**B**)). To model the anisotropic property of hyperelastic material, the reinforced fiber may be embedded in the ground Mooney–Rivlin matrix. The strain energy of the material is formulated as

$$W=C_{1}\left(I_{1}-3\right)+C_{2}\left(I_{2}-3\right)+F\left(\lambda \right)+\frac{1}{2}{\left[Kln\left(J\right)\right]}^{2}$$ (4)

Here, $C_{1}$ and $C_{2}$ are the Mooney–Rivlin coefficients; F(λ) is the function to depict the mechanical properties of axonal fibers; and K is the effective bulk modulus of the material. The fiber is assumed to be unable to resist compressive loading. The strengthening of the fibers is described by an exponential function when the fiber is stretched. As the elongation of fiber exceeds a critical fiber stretch level $\lambda^{*}$ , the behavior of the fibers is depicted by a linear function. The detailed expressions for F(λ) is presented as

$$\frac{\partial F\left(\lambda \right)}{\partial \lambda }=\{\begin{array}{c} 0\;\;\;\;\;\;\;\;\;\;\;\;\;\;\;\;\;\;\;\;\;\;\;\;\;\;\;\;\;\;\;\;\;\;\;\;\;\;\;\;\;\;\;\;\;\;\lambda <1\\ \frac{C_{3}}{\lambda }\left[e^{C_{4}\left(\lambda -1\right)}-1\;\right]\;\;\;\;\;\;\;\;\;\;\;\;\;\;\;\;\;\lambda <\lambda^{*}\\ C_{5}+C_{6}/\lambda \;\;\;\;\;\;\;\;\;\;\;\;\;\;\;\;\;\;\;\;\;\;\;\;\;\;\;\;\;\;\;\lambda >\lambda^{*}\; \end{array}$$ (5)

The spatial direction of the principal orthonormal vector ${\hat{n}}_{\alpha }$ may be expressed as the product of the rotation matrix **R** and the principal referential orthonormal vector ${\hat{N}}_{\alpha }$ , i.e., ${\hat{n}}_{\alpha }=R{\hat{N}}_{\alpha }\;(\alpha =1,2,3$ ). If the initial and current direction for reinforced fiber is correspondingly denoted by the unit vector $a_{0}$ and the unit vector a , then the connection between them is shown as

$$\lambda a=F\cdot a_{0}$$ (6)

where λ is the length of the fiber. Thus, one additional invariant is introduced as

$$I_{4}=a_{0}\cdot \left(Ca_{0}\right)$$ (7)

The resulting nominal stress tensor is given by

$$S=2W_{1}F^{T}+2W_{2}\left(I_{1}I-C\right)F^{T}+2I_{3}W_{3}Fa^{-1}+2W_{4}a_{0}⊗a_{0}$$ (8)

where $W_{i}=\partial W/\partial I_{i}\left(i=1,\;2\ldots ,5\right)\;$ and ⊗ is the tensor product, which may be denoted as ${\left(u⊗v\right)}_{ij}=u_{i}v_{j}$ . If the viscoelasticity is absent, then the stress update is simply described as

$$s_{I}=s;p_{I}=p$$ (9)

where $s_{I}$ and $p_{I}$ , respectively, denote the updated deviatoric stress and pressure. The s and p can be understood as the deviatoric stress and pressure from the last iteration results, respectively. The deviatoric and volumetric decay coefficients $\beta_{s}$ and $\beta_{p}$ are introduced to control the decay of stress

$$s_{I}^{\nabla }=s^{\nabla }-\beta_{s}s_{I};\;\;{\dot{p}}_{I}=\dot{p}-\beta_{p}p$$ (10)

The triangular symbol ∇ and dot ( ⋅ ) in the above equation are the differential operators. The decay coefficients *β*_s_ and *β*_p_ can be defined as constants or calculated by multiplying the decay function with the duration of time step. This anisotropic material model described above was incorporated by a *MAT_SOFT_TISSUE material card coupled with the *ADD_INELASTICITY feature available in LS-DYNA solver (R12.0). The direction of the axonal fiber was defined by applying the *MAT_COORDINATES in each of the white matter structures. As shown in Figures 1D,E, the direction of axonal fiber in the subcortical white matter is scattering-out from the internal capsule to form the corona radiata. The fiber direction was determined by the relative location of elements to the fiber origin that was presumably located above the midbrain in the midsagittal plane. For the corpus callosum white matter structure, the axonal fiber was originated from the medial to the lateral direction in parallel within the anatomical coordinates. For the brainstem, the fibers were directed along the axis direction of the brainstem.

### 2.2 Optimization of Anisotropic Visco-Hyperelastic Material Properties

The strain developed in the brain has been considered as one of the biomechanically relevant indictors of brain injury. Recently, brain displacement data measured from PMHS testing reported by Hardy et al. (2007) was reanalyzed, and strains in the brain were calculated by Zhou et al. (2018). To improve brain strain correlation between the model simulation and experimental results, an optimization study was conducted to find the “best variable” values of the anisotropic materials defined for various brain structures. The optimization was performed by using LS-OPT, which provides an interface with LS-DYNA FE solver to optimize the parameters. The optimization goal was to find a set of property parameters of the anisotropic visco-hyperelastic model whose mechanical response would result in the optimal match to the experimental response. The long-term shear modulus was approximately two times the summation of *C*_1_ and *C*_2_. The stiffness of fiber was determined by *C*_3_, *C*_4_, or *C*_5_. The critical stretch ratio λ^*^ was assumed as 1.06. The bulk modulus (*K*) was calculated from a Poisson’s ratio of 0.49999, simulating incompressible brain tissue (Libertiaux et al., 2011). The corpus callosum and brainstem were assumed to be 25% and 110% stiffer than the gray matter, respectively.

A total of seven PHMS head impact tests conducted by Hardy et al. (2007) were simulated (C380-T1, C380-T2, C380-T3, C380-T4, C380-T6, C288-T3, and C393-T3). The model predicted strain–time histories were compared to the experimental data from all seven cases. The validation score was calculated for each simulation point, and an overall score was calculated based on the weighting factor assigned to each optimization. When the simulation results minimizing the objective function reaches the termination criteria, the LS-OPT will run an additional verification simulation and complete the optimization process. Otherwise, it continues to the next iteration until it reaches the iteration limits.

**FIGURE 2:**
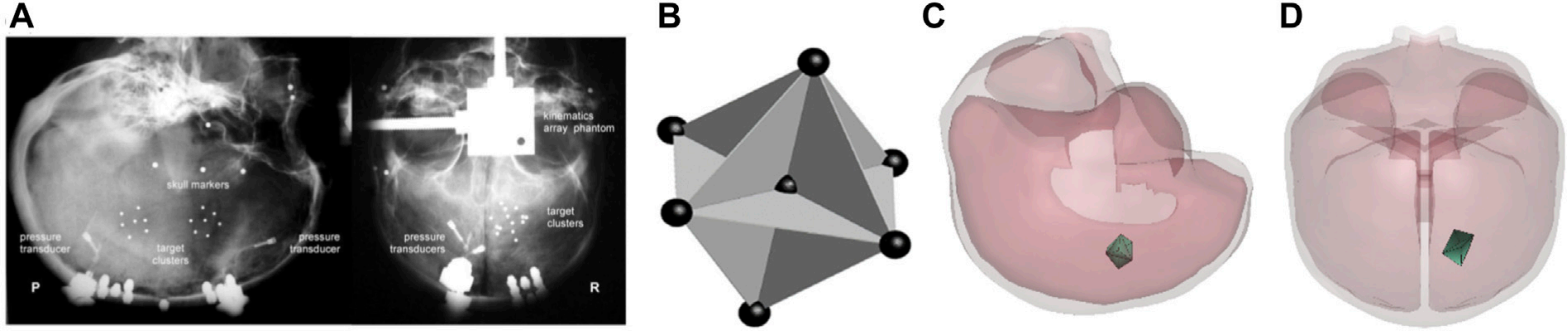
**(A)** The location of NDT cluster in specimen C288 under representative instrumentation x-rays; **(B)** the NDT cluster implanting strategy in the impact tests of Hardy et al. (2007) [this picture is captured from Hardy et al. (2007) and represented with the permission from the Stapp Association]; **(C)** lateral view of null triad model embedded in case C288-T3; **(D)** anterior-posterior view of null triad model implanted in case C288-T3.

Considering both optimization cost and accuracy, two key parameters ( $C_{1}$ and $C_{2}$ ) dominated the brain response and were set as the primary optimization variables per LS-OPT job. The other parameters were optimized subsequently at a given factor to these key parameters. In total, three LS-OPT jobs were completed to optimize $C_{1},C_{2},\;C_{3},\;C_{4}\;$ , and $\beta_{s}$ . The range of the allowable values of the material properties was set according to the published data from material tests of brain tissue (Budday et al., 2019; Chatelin et al., 2010). For each simulation case, seven nodes in the brain model were selected to form 12 null triangular elements (Figures 2A,B) whose coordinates were close to the proximity of the NDTs position implanted in the cadaver brain (Zhou et al., 2018).

The best match between the average Green MPS (GMPS) of the simulated FE triad model and the experimental NDT triad model was considered as the optimal result. The CORelation and Analysis (CORA) (CORAplus 4.0.4, Germany) was used to assess the match of the MPS between the model and experimental results. As presented in Eq. 11, the overall score is calculated with different scale ratings for corridor, phase, magnitude, and shape. A set of parameters that resulted in the highest average CORA score of seven cases was selected as the optimal properties for the brain. Table 1 lists the brain material properties optimized for the GHBMC M50 head model v6.0.

$$S_{overall}=0.4\times S_{corridor}+0.2\times \left(S_{phase}+S_{magnitude}+S_{slope}\right)$$ (11)

**TABLE 1:** Optimized brain material properties defined for the GHBMC v.6.0 brain model.

	RO	$C_{1}$	$C_{2}$	$C_{3}$	$C_{4}$	$C_{5}$	λ^*^	K	*β*_s_
	(g/cm^3^)	(kPa)	(kPa)	(kPa)		(kPa)		(GPa)	
Subcortical White Matter	1.06	−2.19	3.29	1.06	35.6	1.06	1.06	0.154	0.005
Corpus Callosum	1.06	−2.74	4.11	1.60	35.6	1.06	1.06	0.1764	0.005
Brainstem	1.06	−4.57	6.86	1.80	50	2.0	1.06	0.168	0.005
Cortex, Thalamus, Basal Ganglia, Cerebellum	1.06	−2.19	3.29	0	0	0	0	0.154	0.005
CSF, Ventricles	1.04	0.212	0	0	0	0	0	0.034	0.005

RO, density; K, bulk modulus; *β*_s_, bulk modulus decay constant.

### 2.3 Material Properties of Extracranial Structures

The facial and skull bones were simulated with a piecewise linear plasticity material model. The facial flesh and scalp were modeled with the Kelvin–Maxwell viscoelastic material. These two material models and associated properties defined for the new v6.0 model were the same as those defined for all prior versions of the GHBMC head models. The skin was defined with a Kelvin–Maxwell viscoelastic material model in all earlier versions. The skin was updated to an Ogden rubber material for the goal of consistency in the skin model, which was defined for other parts of the body in the GHBMC full body model v6.0. Table 2 lists material parameters and associated properties defined for facial bones, skull, scalp, facial flesh, and skin.

**TABLE 2:** Material properties of the extracranial contents of the head model.

	Density (g/cm^3^)	Young’s (GPa)	Poisson’s ratio	Yield stress (GPa)	Tangent	Failure maximum principal strain
Skull-Tables	2.1	15	0.25	0.09	0.5	0.0088
Skull-Diploe	1	0.6	0.3	0.004	0.02	
Facial Cortical bones	2.1	6	0.25	0.05	0.3	0.0078
Facial Spongy bones	2.1	0.3	0.25	0.006	0.03	
Nose-Septal	2.1	0.02	0.45	0.0002	0.005	0.0078
	Density (g/cm^3^)	Bulk (GPa)	Short-term shear modulus (MPa)		Long-term shear modulus (MPa)	Decay (s^−1^)
Facial Flesh	1.1	0.005	0.68		0.28	3.0e-02
Scalp	1.1	0.02	8.5		3.4	3.0e-02

Skin	Density (g/cm^3^)	*μ*_1_ (MPa)	*μ*_2_ (MPa)	*μ*_3_ (MPa)	g_1_ (MPa)	g_2_ (MPa)	g_3_ (MPa)	g_4_ (MPa)
	1.06	0.6	0.473	-0.14	1.35	24.08	97.73	839.1
	Poisson’s	α_1_	α_2_	α_3_	β_1_	β_2_	β_3_	β_4_
	0.4999	−0.971	−2.393	−3.85	0.00031	0.0167	0.07967	1.246

### 2.4 Model Validation Methods

A total of 34 cases from seven cadaver head impact studies were applied to validate the impact responses of the FE head model in the face, skull, and brain for various structures and regions. The biomechanical response–time histories were compared between the simulation and the experimental results. The methods of validation for each of the parameters are described in Table 3.

**TABLE 3:** Cadaver head impact cases used for the model validation.

Parameters/Structure/Region	Validation parameters	Experiential study
Facial Force: Maxilla and Zygoma Bones	F vs. D	Allsop et al. (1988)
Facial Force: Nasal Bone	F vs. D	Nyquist et al. (1986)
Skull Force: Frontal Bone	F vs. D	Allsop et al. (1988)
Skull Force: Temporal and Parietal Bones	F vs. D	Allsop et al. (1991)
Skull Force: Frontal, Temporal, Occipital, and Parietal Bones	K	Yoganandan et al. (1995)
Intracranial Pressure: Frontal and Occipital	ICP vs. T; peak ICP vs. peak A	Nahum et al. (1977)
Intracranial Pressure: Frontal, Occipital, and Ventricles	ICP vs. T	Trosseille et al. (1992)
Brain/Skull Relative Motion at Specific Locations	D vs. T	Hardy et al., 2001; Hardy et al., 2007
Brain Strain at Specific Locations	MPS vs. T	Hardy et al., 2007; Zhou et al. (2018)

F, force; D, deflection; K, stiffness; ICP, intracranial pressure; T, time; A, acceleration; MPS, maximum principal strain.

#### 2.4.1 Brain Strain Response Validation

TBI occurs when deformations in the neural tissues and cellular components exceed the biomechanical threshold, leading to functional or structural damage. The MPS that is the principal invariant of the strain tensor and its rate are frequently employed as predictive parameters to evaluate the probability of brain injury (King et al., 2003; Zhang et al., 2004; Takhounts et al., 2013; Viano et al., 2005). Hardy et al. (2007) embedded a cluster of NDTs into the PMHS brain tissue to obtain the brain/skull relative motion and strain data during head impacts. Recently, Zhou et al. (2018) revised the method of calculating strain–time histories from the experimental NDT results. Instead of using the relative displacement, the total displacement relative to the initial NDT location was applied as the input to the seven NDTs to deform 12 triad elements formed by NDTs and calculated average GMPS using LS-DYNA (Figure 2).

Seven PMHS head impact cases were simulated by applying experimentally measured 3D head acceleration traces to the head center of the gravity. The brain MPS–time histories validation process was a part of the material optimization process as described in the previous section. Twelve triangular FE elements (null shell elements) formed by seven FE nodes located at locations approximating the initial NDTs inserted in the cadaver brain were defined. The resulting GMPS from the 12 elements were plotted and averaged. Figure 2 shows an example of a triad defined in the FE model.

#### 2.4.2 Intracranial Pressure Validation

Nahum et al. (1977) conducted a series of frontal head impact tests to measure the dynamic intracranial pressure in the brain of pressurized PMHS. The impactor mass ranged between 5.23 and 23.09 kg and impacted the forehead with its Frankfort plane inclined 45° from the horizontal plane. The impact velocity ranged between 8.41 and 12.95 m/s. Pressure sensors were used to measure coup pressure at the impact site in the frontal area, and the contrecoup pressure at the posterior fossae on the opposite side of impact. Six tests (36, 37, 38, 43, 44, and 54) were simulated to validate the intracranial pressure response of the head model v6.0. The acceleration–time history was applied to the center of gravity of the head model. The impact acceleration–time histories of the head were only available for Test 37. The acceleration loadings of the other five cases were scaled from that of Test 37 by using the reported peak magnitude ratio.

Trosseille et al. (1992) carried out a series of PMHS impact tests to measure the intracranial and ventricular pressure. In their study, a 23.4-kg impactor was used to impact the facial region at a speed of 7 m/s in the antero-posterior direction. The intracranial pressure in the frontal and occipital lobes and the ventricular pressure in the lateral and third ventricles were measured. The MS428_2 test was simulated by prescribing experimentally measured head acceleration–time traces to the center of gravity of the head model. The intracranial pressure vs. time history responses predicted by the head model v6.0 at the corresponding locations were compared to the experimental results.

#### 2.4.3 Facial Response Validation

Nyquist et al. (1986) used 25-mm diameter rigid cylindrical impactors to impact the nasal region of the PMHS face along an anterior-to-posterior direction with impact velocities ranging from 2.8 to 7.2 m/s. The impactors weighed 32 or 64 kg. The reported contact forces were calculated from the deceleration and mass of the impactor, and the penetration was calculated *via* the double integration of acceleration signals. The force–penetration curves of four tests (20, 29, 34, and 42) were used to validate the nasal impact response of the head model.

Allsop et al. (1988) performed a series of facial impact tests on PMHS. A 14.5-kg semi-circular rod with a 20-mm-diameter impactor was dropped from a height ranging from 3.05 to 6.1 m for impacts on the forehead, zygomatic, and maxillary regions of the face with its circular surface. A set of load cells was incorporated into the impactor to measure the interface force–time histories while the impactor was penetrating the head. The fracture force for each impact test was detected and identified using both force–time curves and the acoustic emission monitoring method. Force–deflection (F-D) curves reported from six tests were used to validate the model response in the zygomatic and maxilla region, respectively.

To validate the facial response of the model against the test results reported from these two facial impact studies, the FE impactor models of a 14.5-kg semi-cylindrical and a 32- or 64-kg cylindrical impactor were developed. The impactors were defined as rigid body with an elastic material and given initial velocities according to the values used in the tests. The FDs predicted from the model simulations were then compared to the experimental results.

#### 2.4.4 Skull Response Validation

A total of eight skull impact cases from three PMHS experimental studies were simulated to validate the skull responses of the head model v6.0. The first experimental study on PMHS head impact response was reported by Allsop et al. (1991) who investigated the dynamic FD response of the human temporal and parietal bones to impact. In this study, a 12-kg flat rectangular plate (5 × 10 cm) was dropped from a height of 102 cm onto the parietal-temporal region of the skull, and a circular impactor (10.6 kg) with a 2.54 cm diameter was dropped from a height of 38 cm onto the temporal region. The second experimental study was reported by Allsop et al. (1988) who conducted skull impact in the frontal region, the same test series used for the facial impact validation described under the facial validation section. The third experimental study was reported by Yoganandan et al. (1995) who impacted the skull at the rate of 7.1–8.0 m/s with a 96-mm-diameter hemispherical Anvil impactor. The tests were divided into frontal-angled, occipital, and vertical impacts.

The FE models of impactors with semi-cylindrical, circular, and rectangular shapes were developed and used to simulate impacts to different skull bones at various angles and initial speeds of the PMHS experiments. The contact FD histories predicted by the model were compared to a series of experimental results at each of the impact locations (Allsop et al., 1988, 1991). The FD curves were not available from Yoganandan et al. (1995). Instead, the FD slope calculated from the model was used to compare the reported experimental data.

### 2.5 Development of Crash-Induced Injury Indices for Predicting Various Head Injuries

Table 4 lists the 32 experiments from four different studies that were simulated by the head model to develop CIIs for predicting various brain injuries including cerebral contusion, ASDH, and diffuse brain injury. The coup ICP was proposed as a measure for contusion injury. The strain in the BV was applied as a measure for predicting ASDH due to the rupture of the BV in the dura space. The MPS and the product of MPS and strain rate (MPS × MPS rate) in various white matter structures were the measures for diffuse brain injury. These biomechanical response parameters were correlated to the incidence of actual injuries observed in the experiments or field injury data.

**TABLE 4:** Cadaver test studies used to determine the CIIs.

CIIs	Response parameter	Case	Experiential study
Cerebral Contusion	ICP in regions of interest	8 injuries and 5 non-injuries	Nahum et al., 1977;Nahum and Smith. (1976)
Acute Subdural Hematoma	Strain in bridging veins	6 injuries and 9 non-injuries	Depreitere et al. (2006)
Diffuse Brain Injury	MPS, MPS × MPS rate	4 injuries	Franklyn et al. (2005)

To develop CIIs for cerebral contusion and ASDH, one logistic regression model and three parametrical survival analysis models (Weibull, Log-normal, and Log-logistic) were constructed to assess the ability of these proposed parameters in predicting the injuries and to develop the best injury risk functions. The injury probability distribution functions can be defined as follows:

$$\begin{array}{c} Logistic:\;\;\;\;\;\;P\left(t\right)=\frac{1}{1+e^{-\left(\kappa t+\delta \right)}}\\ Weibull:\;\;\;\;\;P\left(t\right)=1-e^{-{\left(\frac{t}{\lambda }\right)}^{\rho }},\;\;\;\;\lambda >0,\;\rho >0 \end{array}\;$$ (12)

In the Weibull survival analysis model shown above, the λ (scale) parameter represents the time when 63.2% of the samples has failed, and the ρ (shape) parameter defines the shape of risk curves.

#### 2.5.1 Cerebral Contusion CII Determination

Nahum and Smith (1976) conducted a set of blunt head impact studies to the frontal head of the repressurized PMHS to investigate the injury limits of vascular hemorrhage. The head acceleration and the pathologic studies of brain contusion were available in the study. A total of eight contusion and five non-injury cases were simulated from the studies by Nahum et al. (1977) and Nahum and Smith (1976). ICPs were analyzed using logistic regression and survival analysis to determine CII for predicting cerebral contusion.

#### 2.5.2 Acute Subdural Hematomas CII Determination

A traumatic ASDH is one of the common rotational acceleration–induced brain injuries in fatal road traffic accidents. Depreitere et al. (2006) performed several occipital impacts causing head rotation in the sagittal plane with 10 PMHS to determine the tolerance of ASDH. After each impact test, a fluoroscopy apparatus was applied to detect the rupture of the cadaver’s BVs. If no rupture was observed, then a second impact with increased severity was delivered and BV rupture was checked. The head acceleration histories of cadaver no. 9 impact test were available. The acceleration histories for the other 14 tests were scaled from the acceleration histories of cadaver no. 9 impact test using the reported magnitude and duration ratios. The head acceleration histories of the 15 cases were used as input to the head model to simulate all 15 tests. The BV strain in the subdural space was calculated, and the CII for ASDH was further developed using logistic regression and survival analysis models.

#### 2.5.3 Diffuse Brain Injury CII Determination

Real-world crash accidents with a range of different levels of head injuries were selected for investigation by Franklyn et al. (2005). Four crashes with AIS 2, AIS 4, AIS 5, and MAIS 5 were reconstructed by using vehicles with similar vehicle models and years. One AIS 0 case was reported originally, but further evaluation indicated that the subject had a brief concussion and assumed as AIS 2 in the current study. All AIS 4+ injuries involved high HIC’s, consistent with the severe brain injuries predicted by the Head Injury Risk Curve of Prasad and Mertz for AIS4+ brain injuries (Mertz et al., 1996). In this work, diffuse axonal injuries cannot be confirmed due to the absence of CT scans and MRI. However, diffuse brain injuries can be diagnosed in AIS 4, AIS 5, and MAIS 5 cases. The three translational and three rotational accelerations measured from the crash test dummy were utilized as loading input to the head model. Because of the limited number of diffuse brain injury reconstruction cases, the averaged results from three AIS 4+ injury cases were used to determine the CII values for diffuse brain injury based on MPS and the MPS × MPS rate from regions of interest in the brain.

## 3 Validation Results

### 3.1 Validation of Brain Strain

**FIGURE 3:**
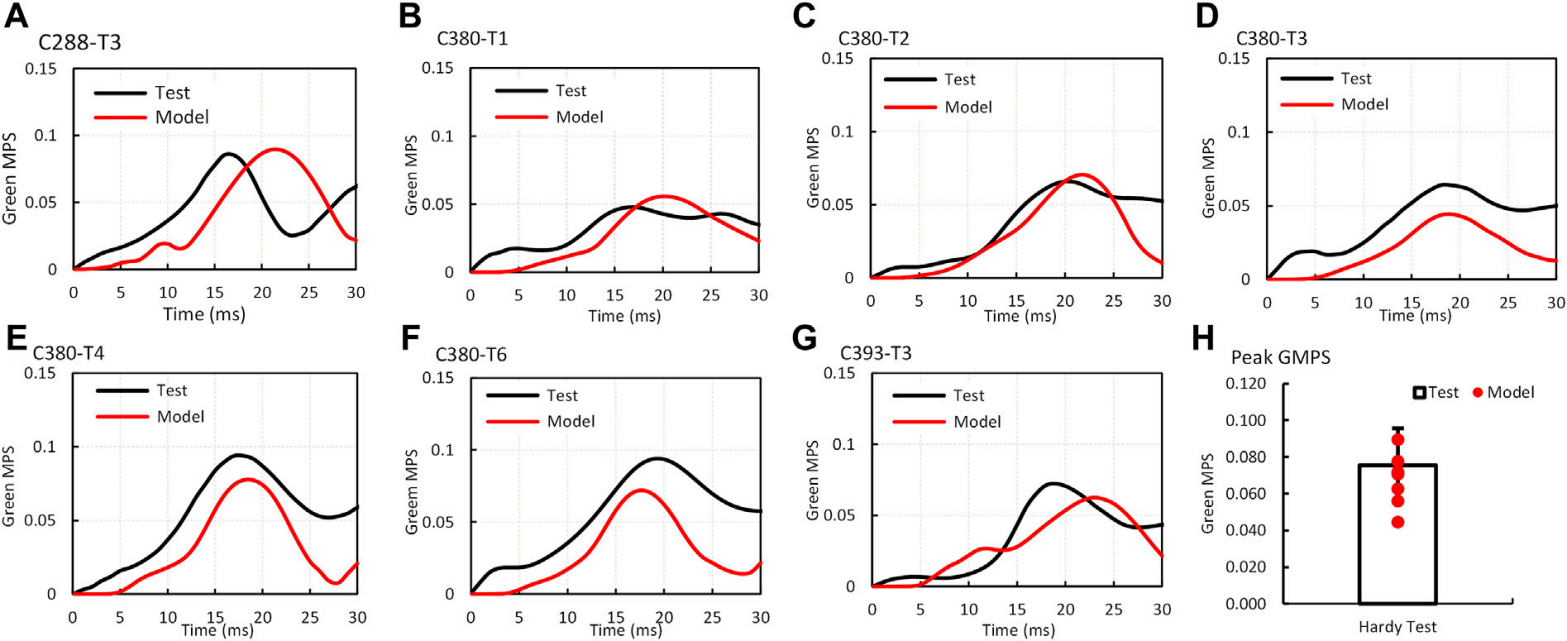
Comparison of Green MPS–time histories and peaks between the GHBMC model simulation and PMHS test **(A)** case C288-T3; **(B)** case C380T1; **(C)** case C380T2; **(D)** case C380T3; **(E)** case C380T4; **(F)** case C380T6; **(G)** case C393T3; **(H)** comparison of peak Green MPS.

The validation process of MPS–time histories in the brain was a part of the material optimization process. The optimization on all seven cases yielded the best correlation of the MPS between the simulations and experiments. The average GMPS histories from 12 triangular elements at one location from each case are shown in Figures 3A–G. Overall, the simulated curves matched well with the experimental curves, especially the trend. The average strain from all seven cases is shown in Figure 3H. The peak MPS of the experiment and model were 0.076 ± 0.02 and 0.067 ± 0.015, respectively. The CORA rating score ranged from 0.48 (C380-T3) to 0.74 (case C380-T2) with an average score of 0.58.

### 3.2 Validation of Intracranial Pressure

**FIGURE 4:**
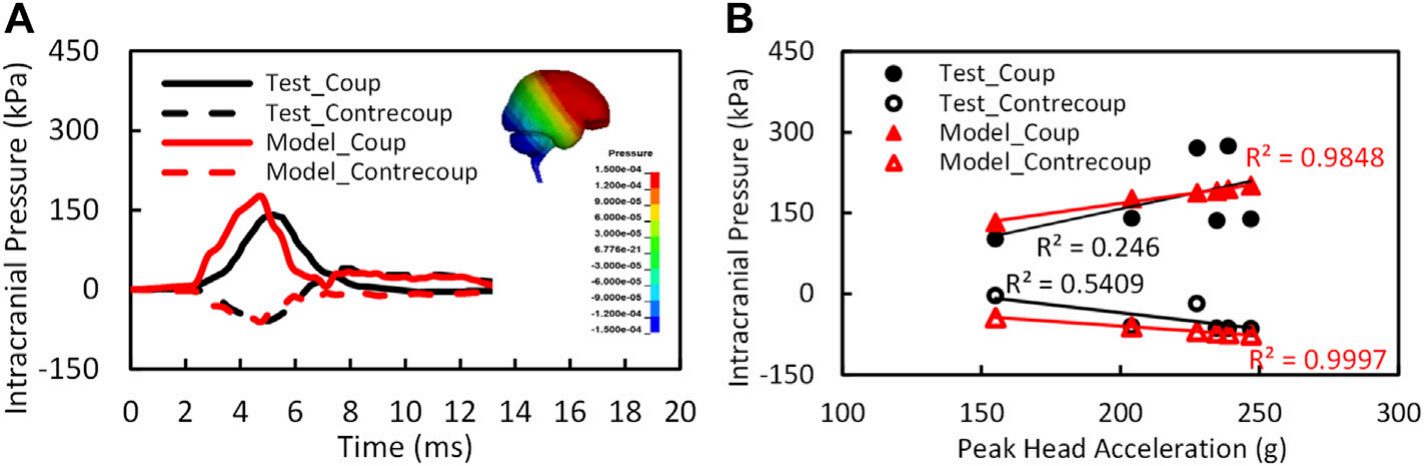
**(A)** The intracranial pressure validation results at coup and contrecoup sites for test 37 (Nahum et al., 1977); **(B)** the relation of intracranial pressures and peak head accelerations (*n* = 6).

Figure 4A shows the model predicted positive intracranial pressure–time histories or compression at the site of impact (coup) and the negative pressure–time histories or tension at the site opposite to the position of impact (contrecoup) in comparison to the experimental data (Test 37 by Nahum et al., 1977). The reported coup pressure reaches its peak at approximately 4.5 ms, slightly ahead of the experimental result. The predicted peak ICP was 176 kPa as compared to 141 kPa from the experiment.

The peak coup and contrecoup pressures for a total of six cases from the simulation and experimental results are plotted with respect to the measured translational head acceleration as shown in Figure 4B. Strong linear relationships were found between the model predicted peak coup/contrecoup pressure and the experimental measured peak head acceleration. However, for the experimental data, the ICP did not show a clear relationship with the head acceleration from six PMHS tests.

The CORA overall score was 0.60 for coup pressure and 0.63 for contrecoup pressure. The phase of the simulated pressure curves showed the highest match to the test, whereas the magnitude score was the least. In general, the model predicted higher pressure than the pressure measured by the sensors in the cadaver brain. There is a possibility that the under-pressurization of the cadaver during preparation might have contributed to lower pressure measurements in the test.

**FIGURE 5:**
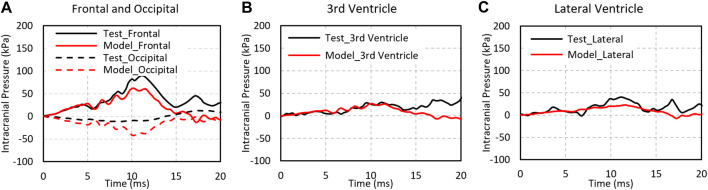
The pressure comparison of test and simulation data for Test No. MS428_2. **(A)** Intracranial pressure at the fontal and occipital regions; **(B)** intracranial pressure at the third ventricle; **(C)** intracranial pressure at the lateral ventricle.

Figure 5 shows the ICPs in the frontal, occipital, third ventricle, and lateral ventricle predicted by the model and experimental results at the corresponding sites (Trosseille et al., 1992). The pressures peaked at approximately 12 ms at all locations and were consistent between the model and the experiment. The ICP predicted in the frontal, third ventricle, and lateral ventricle were 61.6, 27.2, and 22.6 kPa, comparable to 88.6, 30, and 40.6 kPa from experiments at the respective locations. The peak negative pressure predicted by the model at the occipital region was −11 kPa as compared to −41 kPa in the experiment.

### 3.3 Validation of Facial Response

**FIGURE 6:**
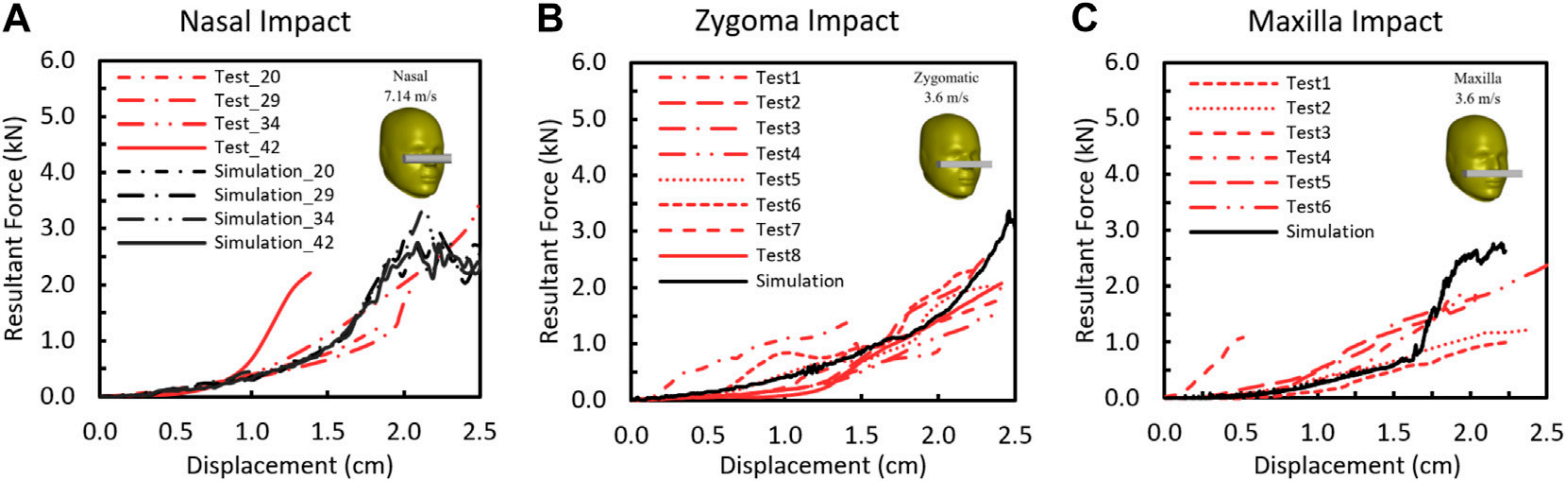
Force–deflection responses between the model prediction and the experimentally measured results for **(A)** nasal bone, **(B)** zygomatic bone, and **(C)** maxilla.

The comparisons of the F-D responses between the simulation and test in nasal, zygomatic, and maxilla regions are shown in Figure 6. The shape of the model predicted facial F-D response to impact showed a concave shape that was consistent with the experimental response. In the test curves, two slopes were observed. The first 4-mm slope denotes the compression of the rubber skin, and the slope between 0.5- and 1.4-cm displacement where the slope is approximately linear was defined as the stiffness/compliance. The simulated F-D curves fell well within the corridor of the experimental results. The stiffness of the model from nasal impact was 0.084 N/m, which is comparable with experiment results (0.1417 ± 0.0649 kN/mm (average ± SD), N = 4). For zygomatic impact, the stiffness of the model response was 0.096 kN/mm, which also fell well within the experimental data (0.09–0.23 kN/m, N = 8). Last, the model stiffness of maxilla impact was 0.22 kN/mm, which falls within the corridor of the reported experimental results (0.08–0.25 N/mm).

### 3.4 Validation of Skull Response

**FIGURE 7:**
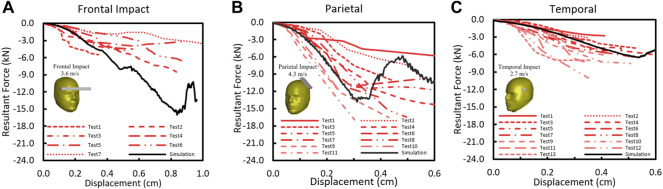
**(A)** Force–deflection response in skull frontal bone (Allsop et al., 1988); **(B)** force–deflection response in skull temporoparietal bone (Allsop et al., 1991); **(C)** Force–deflection response in skull temporal bone (Allsop et al., 1991).

The F-D comparison of skull impacts between the simulation and the test (Allsop et al., 1988, 1991) is summarized in Figures 7A–C. The F-D responses from the model were within the corridor of the test data from frontal bar impact, temporal circular disc impact, and temporal-parietal rectangular plate impact. For the frontal impact, the model stiffness was about 1.9 kN/mm and fell within the range of experimental stiffness from 0.4 to 2.2 kN/mm (Figure 7A). As shown in Figure 7B, the parietal stiffness from the model was 5.5 kN/mm and fell within the reported average value (4.17 ± 1.63 kN/mm). The temporal stiffness was 1.48 kN/mm (Figure 7C), which is consistent within the reported average experimental stiffness 1.8 ± 0.88 kN/mm.

For five experimental cases simulated (Yoganandan et al., 1995), only the experimental stiffness values were available for comparison. The predicted skull model stiffness was 4.8 kN/mm for front impact (45° front_08); 2.5 kN/mm for rear impact (35° rear_10); and 4.09, 4.15, and 4.25 kN/mm for three crown impacts (top_07, top_09, and top_12). The reported experimental stiffness was 5.88, 3.46, 4.80, 2.54, and 4.40 kN/mm, respectively.

## 4 Development of the Crash-Induced Injury Indices

### 4.1 Facial and Skull Fracture CIIs

To determine the proper failure strain and stress limits capable of predicting fracture in the cortical bone and spongy bone by the head model, failure values specified *via* *MAT_ADD_EROSION was tested to initiate the bone fracture at the force level matched to the experimentally results. The simulated facial and skull impact tests were from the PMHS validation case previously mentioned (Nyquist et al., 1986; Allsop et al., 1988; Allsop et al., 1991; Yoganandan et al., 1995).

**FIGURE 8:**
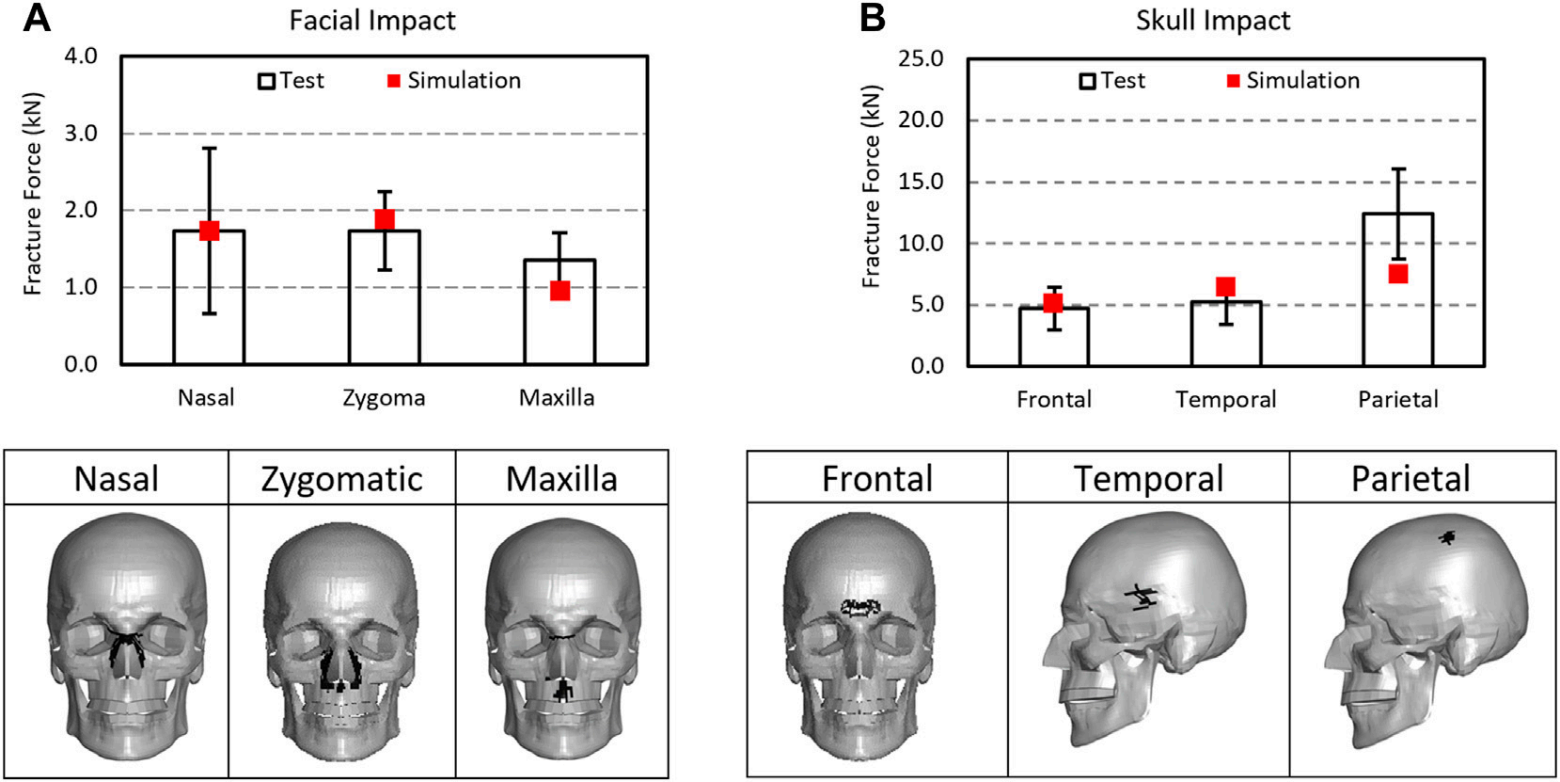
Comparison of fracture forces between the simulations and experiments: **(A)** facial impact and **(B)** skull impact along with fracture predicted in skull and facial bones as illustrated by the removal of the failed elements from the structures.

The contact force at the first failing timepoint reported in the message file was considered as the fracture force for the simulation. The fracture force in the facial bones predicted by the model were 1.78 kN for nasal impact, 1.88 kN for the zygomatic impact, and 0.97 kN from the maxilla impact. The fracture levels by the model fell well within the range of experimental results (1.734 (±0.438), 1.35 (±0.356), and 1.737 (±0.504) kN) in the corresponding facial impact locations. Figure 8 shows the fracture forces from the model simulation and the experiments. The MPS at 0.0078 and MPSS at 20 MPa were determined to be the facial fracture CII parameters and values for cortical and spongy bones, respectively.

For skull fracture prediction, the model predicted fracture forces for frontal, temporal, and temporal-parietal bones were 5.09, 6.39, and 7.52 kN, respectively. These results fell well within the range of the experimental data 4.715 (±1.735), 5.195 (±1.801), and 12.39 (±3.654 kN) in the corresponding regions. The MPS at 0.0088 and MPSS at 20 MPa were determined to be the skull fracture CII values for tables and diploe layers, respectively.

### 4.2 Cerebral Contusion Injury CII

**FIGURE 9:**
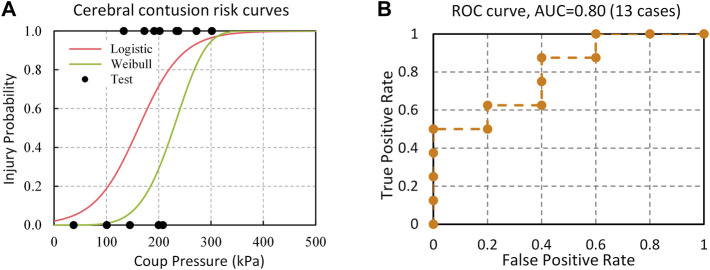
**(A)** Injury risk curves of cerebral contusion based on Logistic regression and Weibull survival analysis models; **(B)** ROC curve of contusion injury predictive data.

The risk functions of cerebral contusion based on logistic regression (Eq. 13) and Weibull survival analysis (Eq. 14) models are given as

$$P{Contusion}_{Logistic}=\frac{1}{1+e^{-0.02394\times coup+3.8606}}$$ (13)

$$P{Contusion}_{Weibull}=1-e^{-{\left(coup/244.92\right)}^{5.29}}$$ (14)

the contusion injury risk models using coup pressure as an injury predictor are shown in Figure 9. The logistic regression and survival analysis models are developed by using the scikit-learn (version 0.24.1, https://scikit-learn.org/stable/) and lifelines (version 0.26.0, https://lifelines.readthedocs.io/en/latest/) python libraries, respectively. The predictive ability and accuracy of logistic regression and the Weibull survival model were assessed by receiver operating characteristic (ROC) curves and the area under the ROC curve (AUC). The ROC curve depicts the compromising relationship between true positive rate (sensitivity) and false positive rate (1-specificity) for all possible cutoff points. The AUC represents the overall accuracy of the model with a value approaching 1.0 indicating a high sensitivity and specificity. The values of AUC were 0.8 for both survival analysis and logistic regression models (Figure 9). As presented in Eq. 15, assessment metrics including accuracy, sensitivity, specificity, and precision were defined using the combination of All Predictions (AP), True Positive (TP), True Negative (TN), False Positive (FP), and False Negative (FN).

Accuracy=(TP+TN)/AP;    Precision=TP/(TP+FP);Sensitivity=TP/(FN+TP); Specificity=TN/(TN+FP) (15)

These metrics were then applied to evaluate the predictive ability of the model when the 50% injury risk was set as the cutoff point. The accuracy, sensitivity, specificity, and precision scores of cerebral contusion prediction were 0.77 (Log) vs. 0.69 (Weibull), 0.88 (Log) vs. 0.5 (Weibull), 0.6 (Log) vs. 1.0 (Weibull), and 0.6 (Log) vs. 1.0 (Weibull), respectively. Among all models, the logistic regression model had the highest predictive ability. Hence, the logistic regression model in Eq. 12 was determined to be the peak ICP–based CII for cerebral contusion. The peak ICP of 161 predicted 50% risk of contusion injury and 38 kPa predicted 5% risk of contusion injury.

### 4.3 Acute Subdural Hematomas Injury CII

The stretch ratio of the BV in the subdural space was calculated for 15 cases. The average maximum strain was 0.533 for injury cases (N = 6) and 0.268 for no-injury cases (N = 9). BV strain was used as a potential injury predictor for ASDH. The risk functions for ASDH based on logistic regression (Eq. 16) and Weibull survival analysis (Eq. 17) were shown as follows:

$$P{ASDH}_{Logistic}=\frac{1}{1+e^{-9.184\times stretch\;ratio+4.195}}$$ (16)

$$P{ASDH}_{Weibull}=1-e^{-{\left(stretch\;ratio/0.6181\right)}^{5.3232}}$$ (17)

**FIGURE 10:**
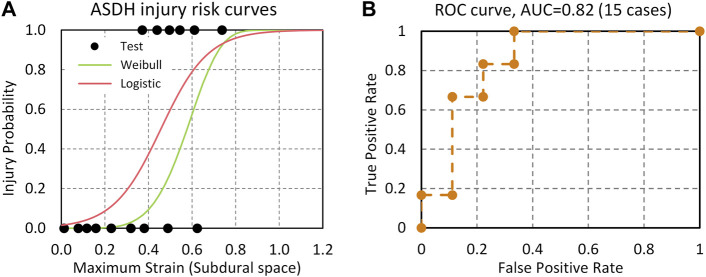
**(A)** ASDH risk curves predicted by the BV strain based on Logistic regression and Weibull survival analysis models; **(B)** ROC curve and AUC value.

Figure 10 shows injury risk curves and actual injury data plotted as functions of the BV strains and ROC curve. The AUC value from the logistic regression model was 0.82. The BV stretch ratio based on subdural space for 50% and 5% injury risk in the logistic regression model was 0.46 and 0.14, respectively. The model assessment metrics were also calculated when the 50% injury risk is set as the threshold. The accuracy, sensitivity, specificity, and precision scores of ASDH prediction are 0.73 (Log) vs. 0.67 (Weibull), 0.67 (Log) vs. 0.33 (Weibull), 0.78 (Log) vs. 0.89 (Weibull), and 0.78 (Log) vs. 0.89 (Weibull), respectively. Overall, the logistic model has the better accuracy and sensitivity than the Weibull survival analysis model.

### 4.4 Diffuse Brain Injury CII

**FIGURE 11:**
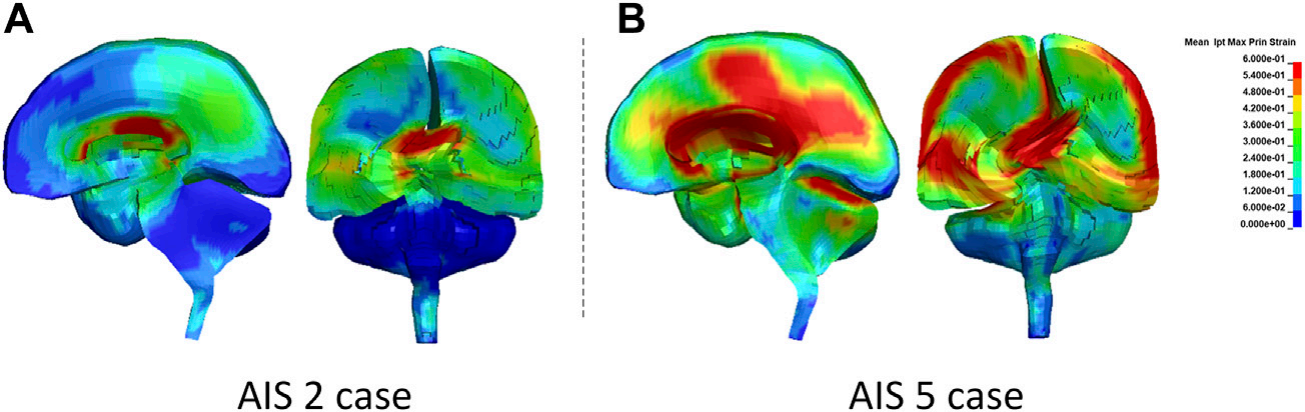
The MPS contour in the sagittal and coronal sections of the brain at the time when the highest MPS occurred: **(A)** AIS 2 case and **(B)** AIS5 case.

The peak MPS magnitude for a given structure/region was represented by an average of the highest MPS from the top 10 elements, instead of taking the highest MPS from one to two elements. It is a common practice to minimize the numerical artifacts of potentially extreme deformation resulting from elements with relatively inferior quality. The average peak MPS from the MPS history curves in the subcortical white matter, corpus callosum, and brainstem structures were 0.66, 0.85, and 0.44 for AIS 2 concussion and 0.69, 0.82, and 0.72 for AIS 4+ diffuse brain injury, respectively. As shown in Figure 11, the corpus callosum showed the larger MPS than other regions.

The peak MPSR was taken from derivatives of MPS histories of the same elements within a given structure. The product of the MPS and MPSR was then calculated by multiplying the MPS and MPSR–time histories. The peak product of the MPS and MPSR for AIS 2 and AIS 4+ cases were 25 and 68 s^−1^ in the subcortical white matter, 34 and 104 s^−1^ in the corpus callosum, and 8 and 146 s^−1^ in the brainstem, respectively. The average MPS, MPSR, and product of MPS × MPSR experienced by the white matter tissues (averaged from all three white matter structures) between AIS 2 and AIS 4+ cases was compared. The comparison revealed that the peak product of MPS and MPSR response in the white matter had superior predictive capability of discriminating AIS 4+ brain injury from AIS 2 concussive injury than MPS response only.

## 5 Discussion

An advanced anisotropic material model was developed, optimized, and incorporated into the revised brain model to improve bio-fidelity of the GHBMC M50 head model v6.0. A comparison study with the old model showed that the anisotropic visco-hyperelastic brain model was more sensitive to the rotational/impact loading directions and could help to discern the injury severities as the loading changed from sagittal to horizontal to coronal of the head. These improvements enable the model to simulate directional properties of the white matter, which helps exhibit more realistic directional dependent impact or rotational response and injury results. The head model v6.0 has been subjected to rigorous validations against a total of 60 PMHS experiments to ensure the accuracy of the computer model in predicting kinematic, kinetic, stress, and strain responses as experienced by the human head from a variety of loading conditions. This validated human head model featured sufficient anatomical details in the skull, face, and brain of various structures, enabling the capability of predicting various injuries and injury localizations. Through the current work, the GHBMC head model is equipped with various injury assessment functions with reference values that were developed from a total of 32 cases with and without observed injury. As shown in Table 5, the GHBMC head model v6.0 has the capability of predicting skull fractures, facial bone fractures, cerebral contusion, ASDH, and diffuse brain injury encompassing injury severities from AIS 1 to AIS 5. Seven of the eight CIIs were ranked at level “0”, the highest capability. Lack of human diffuse brain injury data limited the capability of diffuse brain injury CII at the level “1”, even though the current model has improved material modeling of the white matter tissues.

**TABLE 5:** The injury predictive ability summary of GHBMC FE human head model.

CII description		Capability	Capability comments
Primary	Secondary		
Skull Fracture	Cortical Layer	0	
	Diploe Layer	0	
	Vault	0	
	Base	0	Model detail sufficient, test data available, injury mechanism understood, correlation carried out, and tissue-level CII values are comparable to the literature data
Facial Bone Fracture	Various Facial Bone	0	
Acute Subdural Hematoma	Bridging Vein Rupture	0	
Cerebral Contusion	Cerebral Injury	0	
Diffuse Brain Injury	Cerebrum, Cerebellum, Mid-Brain Injury	1	Model detail sufficient, test data available, injury mechanism understood, correlation carried out, but real-world injury data insufficient

### 5.1 Validation of Brain Strain

The strain magnitude or cumulative strain measures in the brain has been proposed as a biomechanical relevant parameter responsible for diffuse brain injury from *in vivo* experiments and FE simulation of animal and human models (Franklyn et al., 2005; King et al., 2003; Mao et al., 2010; Meaney et al., 1995; Takhounts et al., 2013; Takhounts et al., 2003; Viano et al., 2005; Zhang et al., 2004; Zhou et al., 2018). To the best of the author’s knowledge, only one head model has systematically validated against measured strain data from multiple PMHS head impact tests from all published FE human head models until now (Zhou et al., 2018). The GHBMC head model v6.0 reported here has been validated against the strain responses using seven PMHS tests. The MPS predicted by the current head model showed good agreements with the experimental data. The MPS histories from the model exhibited similar trends to all experimental results. Compared to the average CORA score of 0.4 of the models published by Zhou et al. (2018), the average CORA (v4.4) score of the GHBMC v6.0 is 0.58 when validated against the same dataset. The error of average peak strain (seven cases) compared with the test data was 15% in the current GHBMC v6.0 model and 31% in the head model published by Zhou et al. (2018). Previously, in the GHBMC v5.0 head model in which the material property of the brain was isotropic, the CORA score was 0.52 (Zhang et al., 2019). The incorporation of anisotropic properties in the GHBMC head model v6.0 has improved the strain responses in the deep white matter where the measurements were made in the PMHS subjects.

### 5.2 Force–Deflection Response and Fracture Limits

The predicted fracture forces on the face and skull at different locations were within the range reported in the experimental results. The F-D stiffnesses ranged from 0.084 kN/m to 0.1 kN/mm for impacts to the three facial regions. Overall, the stiffness of the facial bone was found to be lower than that of the skull bone (1.48–5.5 kN/mm) in both experimental and simulation results. Because a different shape and mass of the impactors were used in the skull impact with different loading rates, the loading condition could lead to an increase in brittleness and a decrease in strength under tensile load. As a result, the stiffness of the frontal bone and parietal bone were inconsistent between the cases reported in three studies (Allsop et al., 1988; Yoganandan et al., 1995). However, results from both the simulation and test data indicate that the stiffness of the temporal bone (1.48 kN/mm) seemed to be lower than those of other skull bones due to a much thinner thickness (2–4 mm) in the temporal region.

Results indicate that the compact bone fails in tension whereas the cancellous bone fails in compression. The strain-based failure threshold for the tables of the skull was determined to be 0.88%. The strain limit was like the published failure limits of 0.8% reported by Natali et al. (2008). The maximum principal stress-based failure for the diploe was determined to be 20 MPa, which is within the range of reported failure stress 25.1 ± 13.3 (Evans and Lissner et al., 1957) and 23 ± 6 MPa (Buroah et al., 2013). The pre-set damage levels assigned to the skull and the model predicted forces at failure matched the experimental data.

### 5.3 CIIs for Brain Injuries

The CIIs of the GHBMC M50 head model in all prior versions for predicting cerebral contusion, ASDH, and diffuse brain injuries were based on an average value of the model predictions from the injury cases. In this study, the Weibull survival analysis and logistic regression analysis were conducted to develop risk functions for assessing the probability of cerebral contusion and ASDH. From the logistic regression function, the coup ICP of 161 kPa would suggest a 50% risk of sustaining a cerebral contusion. At coup ICP of 38 kPa, the risk of contusion was under 5%. Ward et al. (1980) investigated the brain intracranial pressure tolerance curve based on the human cadaver head impact test results and proposed that the pressure threshold for moderate brain injuries or contusion was 172 kPa, which indicates 56.6% risk of contusion with the current logistic regression function.

The current model predicts the occurrence of ASDH by monitoring the BV segment average strain in the subdural space until rupture. The logistic regression model based on BV strain from 15 PMHS test cases resulted in an excellent statistical model with a high AUC, precision, and sensitivity. In simulations of the experiments reported by Depreitere et al. (2006), the predicted BV strain of 0.46 was associated with a 50% probability of BV rupture and a BV strain of 0.14 to less than 5% risk of BV rupture. The BV rupture strain value was consistent with the reported failure strain for BVs (0.25–0.50) from the material testing studies (Lee et al., 1989; Monson et al., 2005). Further simulations of real-world human exposures to impact conditions are required to develop an injury risk function associating model responses to risk of ASDH. These exposures might include NFL or real-world event reconstructions by Pellman et al. (2003), Sanchez et al. (2019), and Franklyn et al. (2005) and other fall studies (Doorly and Gilchrist, 2006). In the NFL reconstruction study, angular accelerations higher than 6,000 rad/s^2^ have been estimated in sixteen players with mTBI and obviously not having ASDH. ASDH were not reported in two accident reconstructions reported by Franklyn et al. (2005) in which angular accelerations ranging between 12,000 and 25,000 rad/s^2^ were estimated for two drivers in crashes. Similarly, angular accelerations measured in the caps of boxers exceeded 10,000 rad/s^2^ without any signs of brain injuries.

DAI primarily affects the white matter tracts in the brain. In contrast to the previous versions where global MPS was used, the current CIIs allow for assessing diffuse brain injury *via* using a parameter obtained from injury-specific regions of interest, such as the subcortical white matter, corpus callosum, and brainstem. An injury risk curve could not be created for diffuse brain injury due to the limited test cases. However, an AIS 2 injury case was reconstructed to offer a reference indicating a low risk of diffuse brain injury, and the AIS 4+ injury cases were applied as the criterion indicating a high risk of diffuse brain injury. The stretch ratio of reinforced fiber was applied as the predictor to evaluate the risk of diffuse brain injury. The average MPS, MPSR, and MPS × MPSR of the three white matter structures (cerebral white matter, brainstem, and corpus callosum) were compared between AIS 2 (HIC36 value of 73.25) and AIS 4+ (HIC_36_ values ranging from 2,217 to 6,870) cases. The values indicating low risk of diffuse brain injury are 0.65 for MPS, 61.52/s for MPSR, and 22.43/s for MPS × MPSR. As for the AIS 4+ diffuse brain injury cases, the values are 0.74 for MPS, 213/s for MPSR, and 107/s for MPS × MPSR, respectively. The MPS of an AIS 4+ case is approximately 13% larger than the AIS 2 case, which is a negligible difference. The MPS × MPSR for AIS 4+ is approximately 4.5 times larger than the product of the AIS 2 case. This indicates that the product of strain and strain rate might be a more sensitive injury predictor than the MPS alone. This observation was originally proposed by King et al. (2003) and Viano et al. (2005). They pointed out that strain rate might be a better predictor of diffuse brain injuries than strain.

## 6 Conclusion

In the current work, the anisotropic visco-hyperelastic property of the brain has been developed and incorporated into the upgraded GHBMC M50 human head model. This v6.0 head model improved bio-fidelity of modeling loading direction-dependent response of the white matter tissue under various impact conditions. The optimized material properties have resulted in improved strain predictions in the brain, one of the major injury predictors for TBI. The biomechanical responses from brain strain, intracranial pressure, facial bone, and skull have been validated against multiple PMHS test results. To the authors’ best knowledge, this is the first FE head model that has been rigorously validated against a wide variety of experimental data. Furthermore, multiple CIIs for predicting skull and face fractures, cerebral contusion, ASDH, and DAI have been explored and show improved capability of the current model over previous versions of the GHBMC M50. The authors stated that this tool can be used for testing of various hypothesis advanced as the mechanisms of brain injuries, prediction of injuries and developing countermeasures to reduce risk of injuries in many environments, e.g., falls, transportation, sports and military, etc.

## Data Availability

The original contributions presented in the study are included in the article/Supplementary Material, further inquiries can be directed to the corresponding author.
